# The impact of approaches in improving male partner involvement in the prevention of mother-to-child transmission of HIV on the uptake of safe infant feeding practices by HIV positive women in sub-Saharan Africa. A systematic review and meta-analysis

**DOI:** 10.1371/journal.pone.0207060

**Published:** 2018-12-03

**Authors:** Noah F. Takah, Jeannine A. Atem, Leopold N. Aminde, Moffat Malisheni, Grant Murewenhema

**Affiliations:** 1 Department of Health Policy, International Diagnostics Centre Africa, Addis Ababa, Ethiopia; 2 Department of Clinical Research, London School of Hygiene and Tropical Medicine, London, United Kingdom; 3 Department of Clinical Epidemiology, School of Public Health, University of Queensland, Brisbane, Australia; 4 Department of Maternal and Child Health, Ministry of Health, Lusaka, Zambia; 5 Department of Maternal and Child Health, Ministry of Health, Harare, Zimbabwe; Vanderbilt University Medical Center, UNITED STATES

## Abstract

**Background:**

The low level of male partner involvement in Prevention of Mother to Child Transmission of HIV services such as safe infant feeding practices poses a serious challenge to the implementation of guidelines on safe infant feeding and may undermine efforts towards elimination of mother to child transmission of HIV in sub Saharan Africa(SSA). We conducted a systematic review and meta-analysis to identify the approaches that have been utilized to improve male partner involvement in PMTCT services as well as their impact on the uptake of safe infant feeding practices by HIV positive mothers in SSA.

**Methods:**

In this systematic review and meta-analysis, Ovid Medline, Embase, PsycINFO, Cochrane library, ClinicalTrials.gov, Web of Science and Current Controlled Trials were searched. Only studies performed in SSA that reported an approach that specifically involved male partners and its impact on the uptake of safe infant feeding practices (irrespective of the language and date of publication) were included. Odds ratios were extracted or calculated from studies and combined in a meta-analysis using the statistical package Stata version 11.0. Forest plots were generated using the random effect model.

**Results:**

From an initial 2416 non-duplicate articles, 06 articles were included in the systematic review and meta-analysis. The overall pooled unadjusted OR was 3.08[95%CI: 2.58–3.68], while the effect sizes for interventions aimed at promoting male partner involvement such as verbal encouragement, complex community intervention and enhanced psychosocial interventions were 1.93[95%CI: 1.34–2.79], 3.45[95%CI: 2.79–4.25] and 5.14[95%CI: 2.42–10.90] respectively. Using only adjusted odd ratios, the pooled adjusted OR = 1.78[95%CI: 1.35–2.34]. The I^2^ = 60.1% p = 0.057 using adjusted ORs.

**Conclusion:**

Interventions aimed at promoting male partner involvement such as enhanced psychosocial interventions, verbal encouragement and complex community interventions increase the uptake of safe infant feeding options. The heterogeneity was moderate among studies. More studies including randomised trials that will recruit larger, representative samples of patients are needed in the future.

Prospero registration number: **42016032673**

## Background

### Rationale

The unprecedented commitment of the global fight against mother to child transmission of HIV has resulted in an encouraging reduction in new paediatric HIV infections in sub Saharan Africa(SSA) by 56% since 2010 [1]. However, the disproportionate burden of HIV infection in SSA is still invariably noticed with 220,000 new pediatric HIV infections and 2.6 million children below 15 years living with HIV accounting for nearly 91% of the global burden [1–3]. Contemporary evidence to explain this persistent disproportionate burden point towards the complex socio-cultural context in the region that impedes the effective participation of communities in HIV prevention activities such as safe infant feeding practices by HIV positive mothers [4].

The World Health Organization (WHO) guidelines on safe infant feeding practices for HIV positive mothers calls for exclusive breastfeeding by HIV mothers on antiretroviral therapy or opting out of breastfeeding (infant formula) with discouragement of mixed infant feeding [5]. Despite this recommendation, mixed infant feeding is still largely observed in many settings across sub Saharan Africa mainly due to the sociocultural belief that breast milk might not be sufficient for the infant [6]. Intricately linked to this sociocultural context is the role of male partner involvement in providing the necessary psychosocial support needed by HIV positive women to shun mixed infant feeding [7].

Male partner involvement in the prevention of mother to child transmission of HIV encompasses communication between spouses (including HIV status disclosure), antenatal care attendance and childbirth, antenatal testing and couples combination antiretroviral therapy(ART) and antenatal support during pregnancy and infant feeding decision support [8]. Evidence suggests that majority of women are in favour of involving their male partners in PMTCT. In a study conducted in Malawi to investigate women’s choices with regard to HIV testing, disclosure and partner involvement in infant feeding and care, it was shown that 87% of the women regardless of HIV status felt that partners should be involved in decisions on health care for their child [9]. Furthermore, a systematic review to evaluate the effectiveness of interventions that aim to improve PMTCT service delivery and promote retention throughout the PMTCT steps has shown that the involvement of male partners PMTCT plays a crucial role in retention of pregnant women [10].

Studies in the area of male partner involvement have suggested a positive impact on the uptake of PMTCT services. Baiden *et al* in a cross-sectional study showed that women who demonstrated a strong sense of willingness to be accompanied by their male partners were five times more likely to accept HIV testing during antenatal care (OR 5.2; 95% CI 1.4 to 19.8) [11]. In a prospective cohort study which involved the follow up of 456 pregnant women and their infants in Kenya, Aluiso *et al* showed that vertical transmission risk was lower among women with partner attendance compared with those without [adjusted hazard ratio (aHR) = 0.56, 95% confidence interval (CI): 0.33 to 0.98; P = 0.042] [12]. Furthermore, using a randomised controlled trial (RCT), Kiarie *et al* showed that compliance to antiretroviral regimens in pregnancy was strongly associated with partner notification (OR 7.5; 95% CI 1.4 to 40) [13].

Despite the positive impact of involving male partners, there is evidence to suggest that the level of male partner involvement in PMTCT in SSA is low which may reduce the likelihood of HIV positive mothers adopting safe infant feeding practices and hence undermine the ongoing efforts towards elimination mother to child transmission of HIV(eMTCT) in SSA [14,15]. Studies have reported approaches that can be used in involving male partners in PMTCT such as invitation letters and complex interventions that use more than one strategy to engage male partners in the community [16]. It was therefore necessary to conduct this systematic review and meta-analysis to determine these approaches and their impact on the uptake of infant feeding practices in view of informing policy makers, governments, implementing partners(such as NGOs) and researchers on how the uptake of PMTCT services can be improved through increased engagement of male partners.

### Objectives

1. To determine the interventions/approaches used to improve male partner involvement within the context of PMTCT, specifically safe infant feeding practices by HIV positive mothers in SSA.
2. To determine the impact of the approaches used in (1) on the uptake of safe infant feeding practices by HIV positive mothers in SSA.

## Methodology

### Protocol and registration

This systematic review with meta-analysis was conducted in accordance with the PRISMA (Preferred Reporting Items for Systematic review and Meta-Analysis) statement [17]. The protocol for this systematic review was registered in the international prospective register of systematic reviews (PROSPERO). The registration number is CRD42016032673. The protocol was published in the British Medical Journal (BMJ) Open [18]. In the protocol we considered outcomes such as infant antiretroviral prophylaxis uptake, maternal ART uptake, safe infant feeding practices, condom use as well as family planning. However, we decided in this review to focus on the safe infant feeding practices outcome due to its significant role in reducing the vertical transmission of HIV.

### Eligibility criteria

This review considered studies that were conducted in SSA. Studies conducted outside of the SSA region were excluded. No restriction was placed on the setting of the study and the language of study. Randomized controlled trials, prospective and retrospective cohort studies, and serial cross sectional studies were eligible for inclusion. Studies were included if they provided data on the impact of male partner involvement on the uptake of safe infant feeding practices. One time cross-sectional studies and case-control studies were excluded because they did not present any evidence of the impact of male partner involvement. The participants were HIV positive mothers.

### Information sources and search strategy

A literature search was conducted from December 1st 2015 through May 30th 2018(inclusive). A search strategy was developed by the principal investigator (NFT) with input from Co-authors JAA and LNA using evidence from a United States Centre for Disease Control and Prevention(CDC) study on how to carry out a detailed systematic search in HIV prevention [19]. Six main databases were searched: Ovid Medline, Ovid Embase, Ovid Health and Psychosocial Instruments (HPSI), PsycINFO, Web of Science and Cochrane library. Current Controlled Trials and ClinicalTrials.gov were searched for ongoing and newly completed trials. A final search was conducted on May 30, 2018. A detailed search strategy is shown in Table 1.

**Table 1:** Search strategy: Embase, Medline and HPSI search strategy.

1	HIV.mp. [mp = ti, ab, hw, id, tn, ot, dm, mf, dv, kw, fx, dq, nm, kf, px, rx, ui, sy] (692761)
2	HIV infection.mp. [mp = ti, ab, hw, id, tn, ot, dm, mf, dv, kw, fx, dq, nm, kf, px, rx, ui, sy] (140551)
3	human immuno-deficiency virus.mp. [mp = ti, ab, hw, id, tn, ot, dm, mf, dv, kw, fx, dq, nm, kf, px, rx, ui, sy] (524)
4	human immune-deficiency virus.mp. [mp = ti, ab, hw, id, tn, ot, dm, mf, dv, kw, fx, dq, nm, kf, px, rx, ui, sy] (806)
5	human immunedeficiency virus.mp. [mp = ti, ab, hw, id, tn, ot, dm, mf, dv, kw, fx, dq, nm, kf, px, rx, ui, sy] (39)
6	(human immun* and deficiency virus).mp. [mp = ti, ab, hw, id, tn, ot, dm, mf, dv, kw, fx, dq, nm, kf, px, rx, ui, sy] (1423)
7	AIDS/pc (28493)
8	acquired immune-deficiency syndrome.mp. [mp = ti, ab, hw, id, tn, ot, dm, mf, dv, kw, fx, dq, nm, kf, px, rx, ui, sy] (139594)
9	acquired immunedeficiency syndrome.mp. [mp = ti, ab, hw, id, tn, ot, dm, mf, dv, kw, fx, dq, nm, kf, px, rx, ui, sy] (35)
10	acquired immunedeficiency syndrome.mp. [mp = ti, ab, hw, id, tn, ot, dm, mf, dv, kw, fx, dq, nm, kf, px, rx, ui, sy] (35)
11	HIV.mp. [mp = ti, ab, hw, id, tn, ot, dm, mf, dv, kw, fx, dq, nm, kf, px, rx, ui, sy] (692761)
12	HIV infection.mp. [mp = ti, ab, hw, id, tn, ot, dm, mf, dv, kw, fx, dq, nm, kf, px, rx, ui, sy] (140551)
13	human immuno-deficiency virus.mp. [mp = ti, ab, hw, id, tn, ot, dm, mf, dv, kw, fx, dq, nm, kf, px, rx, ui, sy] (524)
14	human immune-deficiency virus.mp. [mp = ti, ab, hw, id, tn, ot, dm, mf, dv, kw, fx, dq, nm, kf, px, rx, ui, sy] (806)
15	human immunedeficiency virus.mp. [mp = ti, ab, hw, id, tn, ot, dm, mf, dv, kw, fx, dq, nm, kf, px, rx, ui, sy] (39)
16	(human immun* and deficiency virus).mp. [mp = ti, ab, hw, id, tn, ot, dm, mf, dv, kw, fx, dq, nm, kf, px, rx, ui, sy] (1423)
17	AIDS/pc (28493)
18	acquired immune-deficiency syndrome.mp. [mp = ti, ab, hw, id, tn, ot, dm, mf, dv, kw, fx, dq, nm, kf, px, rx, ui, sy] (139594)
19	acquired immunedeficiency syndrome.mp. [mp = ti, ab, hw, id, tn, ot, dm, mf, dv, kw, fx, dq, nm, kf, px, rx, ui, sy] (35)
20	acquired immunedeficiency syndrome.mp. [mp = ti, ab, hw, id, tn, ot, dm, mf, dv, kw, fx, dq, nm, kf, px, rx, ui, sy] (35)
21	(acquired immune* and deficiency syndrome).mp. [mp = ti, ab, hw, id, tn, ot, dm, mf, dv, kw, fx, dq, nm, kf, px, rx, ui, sy] (139603)
22	vertical transmission.mp. [mp = ti, ab, hw, id, tn, ot, dm, mf, dv, kw, fx, dq, nm, kf, px, rx, ui, sy] (21660)
23	vertical infectious disease transmission.mp. [mp = ti, ab, hw, id, tn, ot, dm, mf, dv, kw, fx, dq, nm, kf, px, rx, ui, sy] (17)
24	mother-to-child transmission.mp. [mp = ti, ab, hw, id, tn, ot, dm, mf, dv, kw, fx, dq, nm, kf, px, rx, ui, sy] (9980)
25	Parent-to-child transmission.mp. [mp = ti, ab, hw, id, tn, ot, dm, mf, dv, kw, fx, dq, nm, kf, px, rx, ui, sy] (315)
26	Maternal-to-child transmission.mp. [mp = ti, ab, hw, id, tn, ot, dm, mf, dv, kw, fx, dq, nm, kf, px, rx, ui, sy] (234)
27	maternal-fetal infection transmission.mp. [mp = ti, ab, hw, id, tn, ot, dm, mf, dv, kw, fx, dq, nm, kf, px, rx, ui, sy] (6)
28	MTCT.mp. [mp = ti, ab, hw, id, tn, ot, dm, mf, dv, kw, fx, dq, nm, kf, px, rx, ui, sy] (2205)
29	PMTCT.mp. [mp = ti, ab, hw, id, tn, ot, dm, mf, dv, kw, fx, dq, nm, kf, px, rx, ui, sy] (3188)
30	pPTCT.mp. [mp = ti, ab, hw, id, tn, ot, dm, mf, dv, kw, fx, dq, nm, kf, px, rx, ui, sy] (105)
31	male partner.mp. [mp = ti, ab, hw, id, tn, ot, dm, mf, dv, kw, fx, dq, nm, kf, px, rx, ui, sy] (4232)
32	spouse*.mp. [mp = ti, ab, hw, id, tn, ot, dm, mf, dv, kw, fx, dq, nm, kf, px, rx, ui, sy] (55107)
33	husband.mp. [mp = ti, ab, hw, id, tn, ot, dm, mf, dv, kw, fx, dq, nm, kf, px, rx, ui, sy] (14458)
34	couple*.mp. [mp = ti, ab, hw, id, tn, ot, dm, mf, dv, kw, fx, dq, nm, kf, px, rx, ui, sy] (704937)
35	father*.mp. [mp = ti, ab, hw, id, tn, ot, dm, mf, dv, kw, fx, dq, nm, kf, px, rx, ui, sy] (101077)
36	men.mp. [mp = ti, ab, hw, id, tn, ot, dm, mf, dv, kw, fx, dq, nm, kf, px, rx, ui, sy] (1119631)
37	sexual partner*.mp. [mp = ti, ab, hw, id, tn, ot, dm, mf, dv, kw, fx, dq, nm, kf, px, rx, ui, sy] (36091)
38	infant feeding.mp. [mp = ti, ab, hw, id, tn, ot, dm, mf, dv, kw, fx, dq, nm, kf, px, rx, ui, sy] (16098)
39	breastfeeding.mp. [mp = ti, ab, hw, id, tn, ot, dm, mf, dv, kw, fx, dq, nm, kf, px, rx, ui, sy] (47383)
40	infant formula*.mp. [mp = ti, ab, hw, id, tn, ot, dm, mf, dv, kw, fx, dq, nm, kf, px, rx, ui, sy] (13156)
41	infant bottle feeding.mp. [mp = ti, ab, hw, id, tn, ot, dm, mf, dv, kw, fx, dq, nm, kf, px, rx, ui, sy] (14)
42	11 or 12 or 13 or 14 or 15 or 16 or 17 or 18 or 19 or 20 or 21 (772668)
43	22 or 23 or 24 or 25 or 26 or 27 or 28 or 29 or 30 (29944)
44	31 or 32 or 33 or 34 or 35 or 36 or 37 (1962751)
45	38 or 39 or 40 or 41 (68733)
46	42 and 43 and 44 and 45 (1245)

Database: PsycEXTRA <1908 to May 14, 2018>, Embase Classic+Embase <1947 to 2018 June 05>, Ovid MEDLINE(R) Epub Ahead of Print, In-Process & Other Non-Indexed Citations, Ovid MEDLINE(R) Daily, Ovid MEDLINE and Versions(R) <1946 to May 30, 2018>

The outputs of the search were exported to Mendeley desktop 1.16.1 and duplicates were removed. After removal of duplicates in Mendeley, the titles and abstracts of the studies were screened independently by NFT and JAA. The full texts were obtained from the screened abstracts after inclusion and exclusion criteria were applied. Authors of articles were contacted for further information on any publication.

### Data collection process and data items

A data extraction spreadsheet was developed in excel version 2013. The data extraction sheet captured characteristics of the studies such as: authors, country of study, study design/method, study population, approaches/intervention used for PMTCT improvement, and ORs (odds ratios).

The outcome of interest was uptake of safe infant feeding practices. Odds ratios were extracted from individual studies. Relative risks and proportions were converted to ORs. Two reviewers (NFT and JAA) independently extracted these data from the included studies. Any disagreement was settled by a third reviewer (LNA). The actual number of participants that incorporated/utilized (or did not incorporate/utilize) safe infant feeding practices after the male involvement approaches were extracted from the studies and odds ratios were calculated from a 2x2 table and the 95% confidence interval (CI) of the odds ratios were also calculated. Also, adjusted odds ratios were extracted from the studies. These has been summarized in an excel sheet and submitted as part of the data extraction sheet (S2 Table). The characteristics of included studies were summarized in Table 2.

**Table 2:** Characteristics of included studies.

Author	Study population	Study design	Approach used	Sample size	Odds Ratio(OR)	Reference
Aluiso et al	HIV positive mother attending clinic in Kenya	Cohort	Verbal encouragement	456	1.59(1.20–2.44)(Extracted)	[12]
Brou et al	HIV positive breastfeeding mothers in Ivory Coast	Cohort	Complex community interventions	546	1.54(1.04–2.27)(Extracted)	[24]
Farquhar et al	HIV positive mother attending clinic in Kenya	Cohort	Enhanced psychosocial intervention	122	5.1(1.08–24.05)(Calculated)	[25]
Kalembo et al	HIV positive mothers attending clinic in Malawi	Cohort	Verbal encouragement	162	3.1(1.6–6.2)(Extracted)	[22]
Msuya et al	HIV positive breastfeeding mothers in Tanzania	Cohort	Enhanced Psychosocial intervention	171	5.15(2.18–12.16)Extracted.5.80(2.05–16.26)after adjustment	[23]
Semrau et al	HIV Positive mother attending clinic in Zambia	Cohort	Complex community interventions	2141	4.8(3.74–6.17)Calculated	[26]

### Synthesis of results and data analysis

The studies that remained relevant following application of inclusion and exclusion criteria were used in the synthesis. Studies with data on impact of male involvement on the uptake of safe infant feeding practices were considered for a meta-analysis that was performed using statistical software Stata version 11.0. In this review the studies included varied significantly in terms of approaches and outcomes which suggests that the true effect sizes measured could also differ. These disparities could very likely introduce high heterogeneity. Therefore, the random effect model was used to pool the evidence from the studies.

Heterogeneity was assessed using the I squared statistic generated. Heterogeneity refers to the variation between the included studies and it was assessed as follows: if the I^2^ = 25%-49% we considered a “low” heterogeneity, if the I^2^ = 50%-74% we considered a “moderate” heterogeneity and if the I^2^ ≥75% we considered a “high” heterogeneity [20].

### Quality assessment of studies

The Newcastle Ottawa scale was used in assessing the quality of non-randomized studies [21]. This scale captured 8 core elements divided into 3 broad elements related to the study quality. The first element was to determine the representativeness of the exposed cohort. The second element was to determine if the study controlled for other variables. The Third element was to determine if there was bias in the measurement of the outcome.

## Results

**Fig 1:**
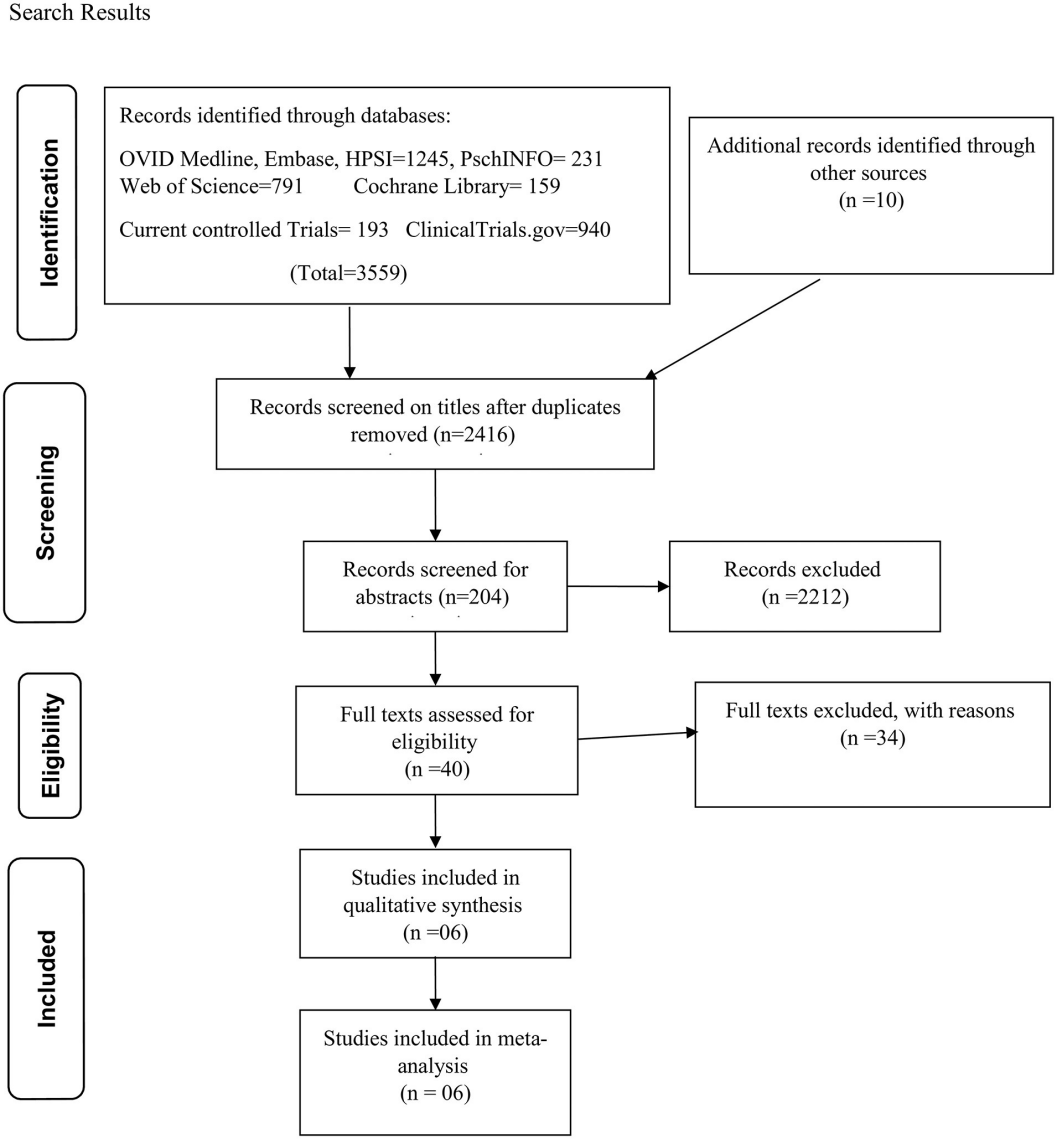
PRISMA flow diagram showing databases searched, screening and inclusion of studies.

The electronic search on Ovid Medline, Ovid Embase, Ovid Health and Psychosocial instruments, Web of Science, Cochrane library, ClinicalTrial.gov, Current controlled trials returned 3559 results and after removal of duplicates this reduced to 2416 results. The study selection process is shown on the PRISMA flow diagram in Fig 1.

Six studies were finally included in the systematic review and meta-analysis. All six studies were cohort studies.

### Characteristics of included studies

Table 2 shows the characteristics of included studies. The studies included a total of 3598 HIV positive mothers ranging from 122 to 2141.

In four of the studies, HIV positive mothers were encouraged verbally through counselling to bring their partners for counselling to the clinic [12,22–24]. Kalembo *et al*, Msuya *et al* and Aluiso *et al* gave no description of the verbal message given to the women or the personnel responsible for providing/delivering the message while Brou *et al* actively encouraged the women through counselling to engage their male partners through HIV status disclosure.

One study by Farquhar *et al* used enhanced psychosocial intervention to involve male partners [25]. It was termed “enhanced” because trained HIV-positive male or female peer counselors were responsible to perform the couples counseling. The couples decided on the gender of the HIV peer counsellor. These specifically trained peer counselors were well equipped with communication skills that could improve on the level of male partner involvement.

The study by Semrau *et al* involved the use of complex community interventions to improve on male partner involvement [26]. These were complex community interventions because several approaches to involve male partners were coupled with other changes in health care delivery to improve on maternal and child care within the community. Community mobilization talks and dramas were carried out. During these community meetings, community members were educated on the relevance of early antenatal clinic attendance of couples and the role of male partners in supporting the uptake of PMTCT services by women. Semrau *et al* engaged community health workers to carry out mobilization talks and dramas in football events, market places, clubs, churches and work places.

### Quality assessment

**Fig 2:**
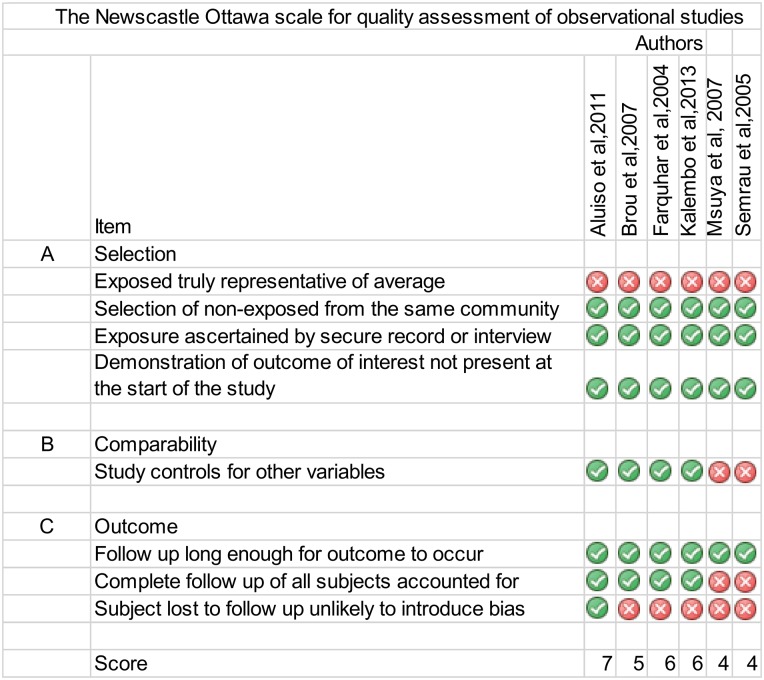
Quality assessment of observational studies using the Newcastle Ottawa scale.

The results of the quality assessment are shown in Fig 2. None of the final eligible studies selected a representative sample of patients which most likely resulted in selection bias. In three studies the cohort possessed special characteristics that did not represent the general characteristics in the setting [23–25]. In the Farquhar *et al* study, the couples were older, more educated and more likely to engage in HIV prevention activities. Brou *et al* recruited a consecutive sample of older HIV positive women who were more likely to be in polygamous marriages. Msuya *et al* included women who were younger, less educated and less likely to engage in prevention activities. Semrau *et al* consecutively selected participants only from socioeconomic disadvantaged communities while Aluiso *et al* and Kalembo *et al* recruited consecutively from a single district hospital.

Two of the studies did not adjust for confounding [23,26]. Furthermore in terms of follow-up, participants in all the final selected studies had long enough follow-up time to observe the outcome but only the study by Aluiso *et al* provided evidence to justify that the characteristics of participants lost to follow-up(LTFU) were similar to those retained [12]. The other studies were prone to follow-up bias because the authors didn’t investigate if the characteristics of patients lost to follow-up were similar to those retained.

### Results of meta-analysis

**Fig 3:**
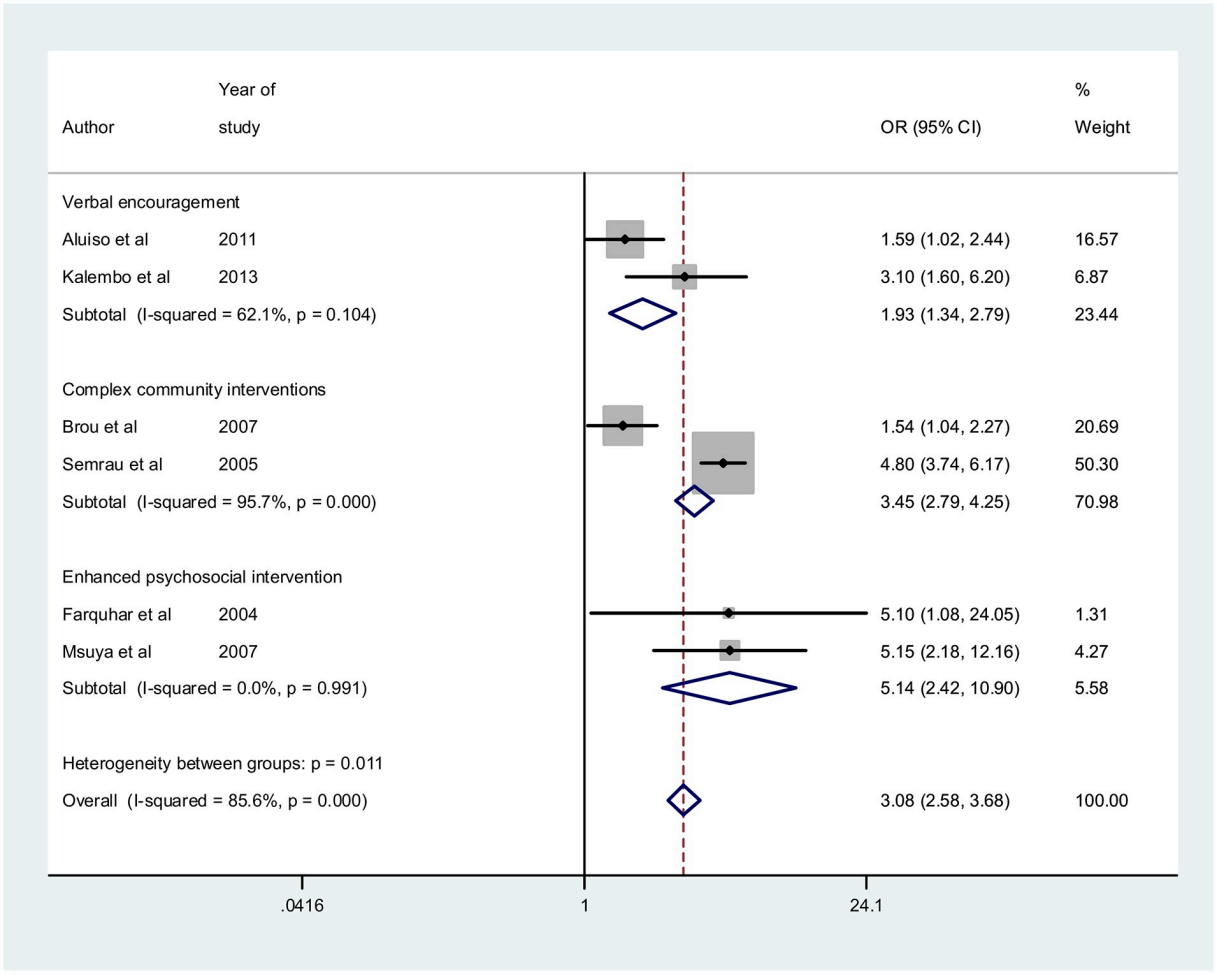
Overall forest plot by categories of approaches for studies reporting the impact on safe infant feeding practices.

**Fig 4:**
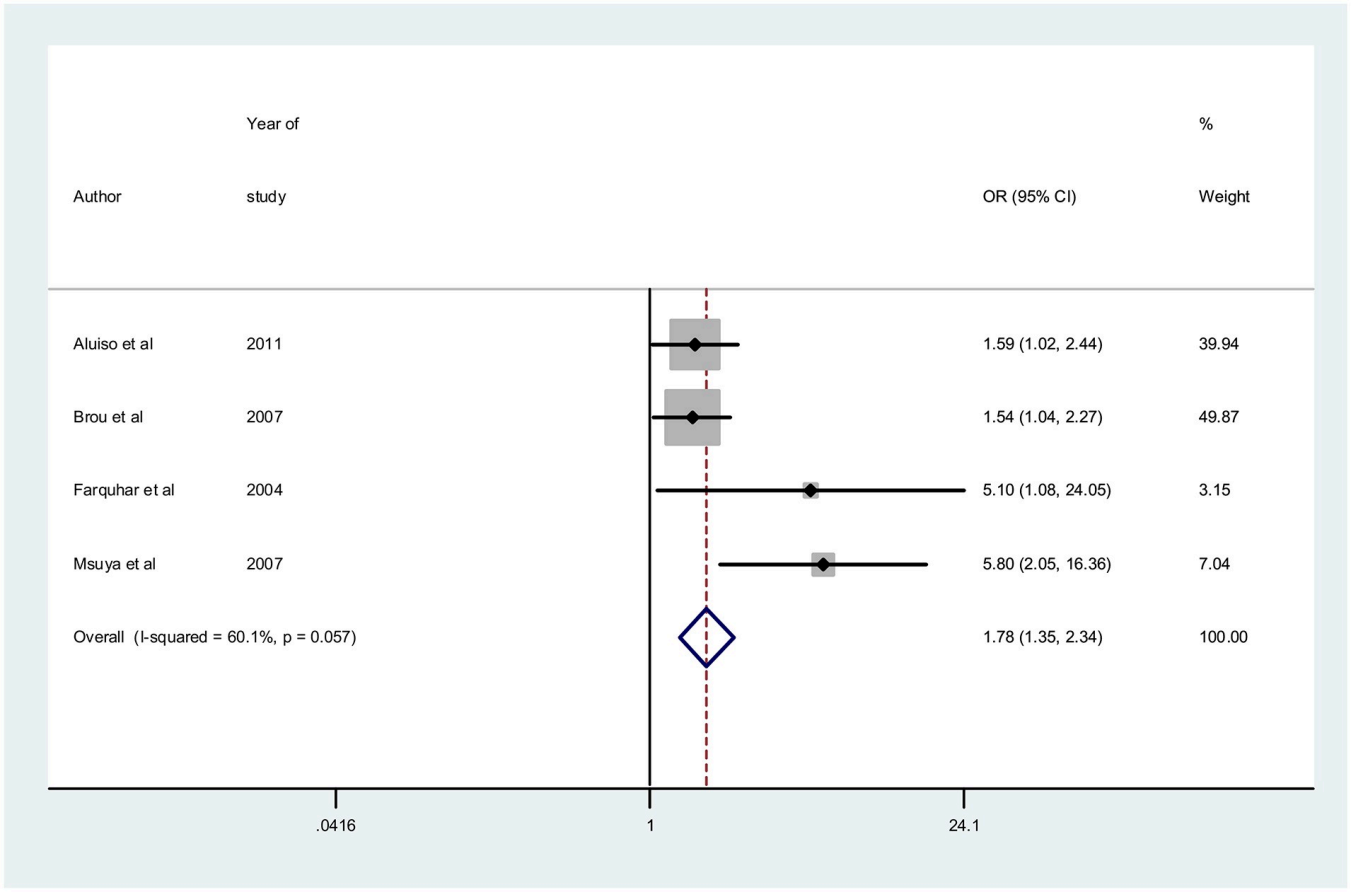
Forest plot for adjusted odds ratios for studies reporting impact on safe infant feeding practices.

**Fig 5:**
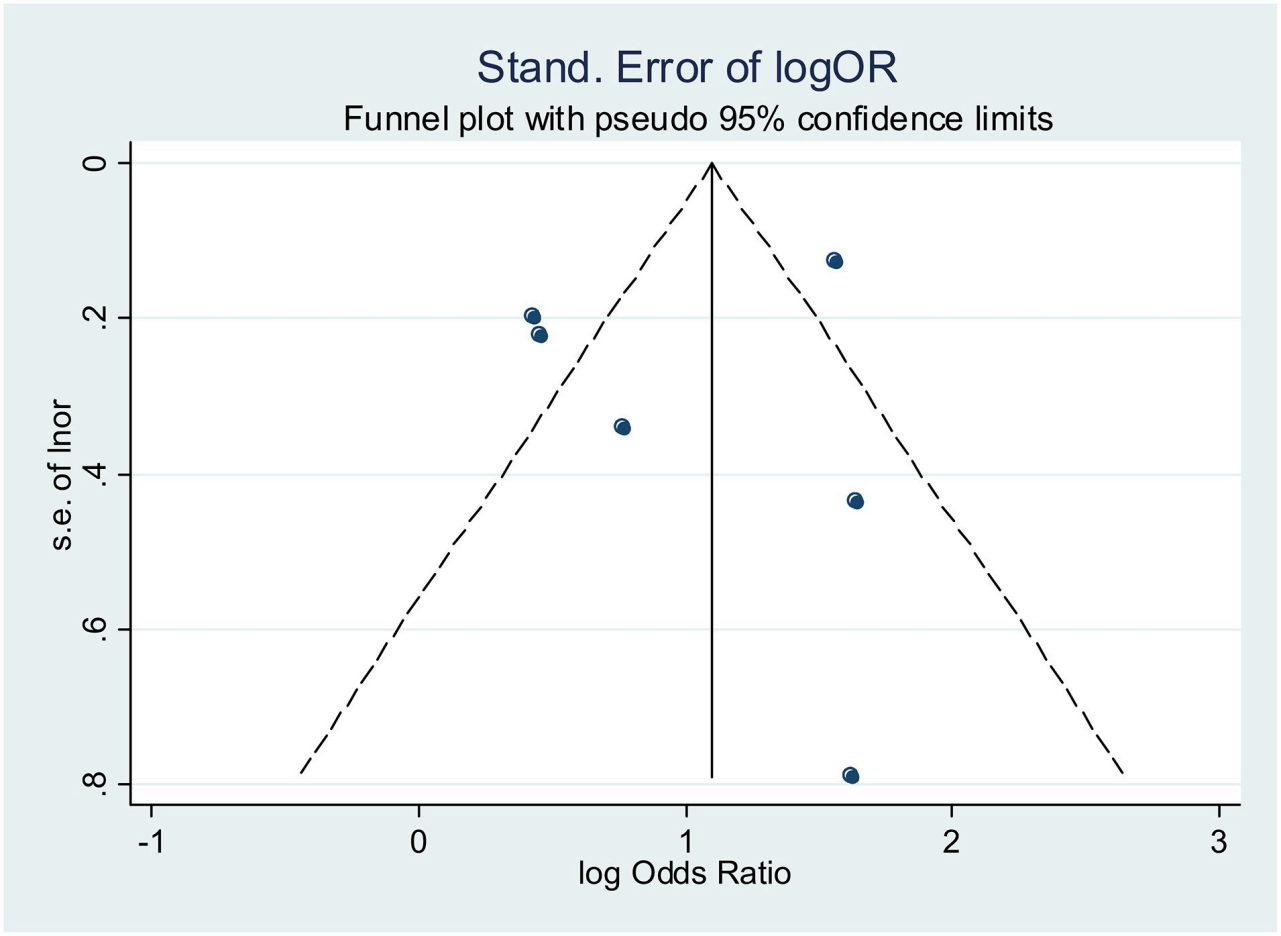
Funnel plot for publication bias for studies reporting impact on safe infant feeding practices.

Fig 3 shows the overall forest plot for the studies that reported the impact of male involvement on the uptake of safe infant feeding practices. The pooled unadjusted OR = 3.08[95%CI: 2.58–3.68] while the effect sizes for interventions aimed at promoting male partner involvement such as verbal encouragement, complex community intervention and enhanced psychosocial interventions are 1.93[95%CI: 1.34–2.79], 3.45[95%CI: 2.79–4.25] and 5.14[95%CI: 2.42–10.90] respectively. In Fig 4, using only adjusted odd ratios, pooled adjusted OR = 1.78[95%CI: 1.35–2.34]. The I^2^ = 60.1% p = 0.057 using adjusted ORs. The funnel plot in Fig 5 shows very a uniform scaterring of points along the central axis.

## Discussion

A meta-analysis of Odds Ratios from six studies showed a statistically significant increase in the uptake of safe infant feeding practices with male partner involvement. This increase was associated with enhanced psychosocial intervention, verbal encouragement and complex community interventions. These findings are in contrast to what Brusamento and colleagues reported [27]. The systematic review by Brusamento *et al* suggested that male involvement had a negative impact on the uptake of PMTCT services by HIV positive women [27]. Even though Brusamento *et al* thoroughly evaluated multiple databases with a comprehensive and systematic search strategy, independently assessing the quality of individual studies, the search strategy was biased towards identifying only randomized controlled trials which might explain why the authors failed to also include relevant cohort studies that could have improved on the evidence synthesis with possible meta-analysis. Another possible reason for the disagreement with the Brusamento *et al* study is that the authors drew their conclusion from a single study that used only invitation letters as an approach to involve male partners. Our study has shown that other categories of interventions exist such as enhanced psychosocial intervention, verbal encouragement and complex community interventions that have a positive impact on the uptake of safe infant feeding practices. In addition, by pooling together the evidence from different studies in a meta-analysis, our study provides more convincing evidence than the Brusamento *et al* study that drew conclusions from a single unblinded randomised trial.

Our study is also different from two reviews conducted in the domain of male involvement [28,29]. The systematic review by Yargawa and colleagues focused only on maternal outcomes such as postpartum depression, utilization of hospital services and postnatal care [28]. The postpartum care did not involve infant feeding practices. Compared with the Auvinen *et al* study [29], our study did not identify invitation letters as a strategy to specifically improve the uptake of safe infant feeding practices. However, it should be noted that the study by Auvinen *et al* was a narrative review with no systematic search strategy, no multiple database search, and no independent assessment of the quality of included studies. In addition, since the Auvinen study was a narrative review with no focus on the impact on outcomes, the authors could not provide data on the impact of male involvement on the uptake of safe infant feeding practices.

Our study has shown that enhanced psychosocial interventions improve the uptake of safe infant feeding practices. The use of HIV-positive peer counselors was an advantage because it appeared to encourage sero-concordant couples to more readily accept their status. Using well trained peer counsellors ensured that they were well equipped with communication skills that could improve on the level of male partner involvement [30]. In addition, allowing the couples to choose the gender of the counselor improved the response rate and overall acceptance of counselling. This acceptance may have a positive impact on the subsequent lifelong behavioural adaptations that could have long-term benefits for the family [31]. However, identifying these HIV-positive peer counselors may be challenging in communities within SSA where stigma and fear of status disclosure still form a major component of the social context [32].

In our study, the use of complex community interventions to improve the uptake of safe infant feeding practices was a strength for many reasons. We feel that four of the most important strengths include the following: 1) The large sample size correlated with robust statistical power to find statistically significant differences not due to chance. 2)By involving communities the authors went closer to the male partner than in other approaches mentioned so far. This could reduce the chances of measurement errors seen in other studies that relied on what was reported from the pregnant women. 3)Utilizing several approaches within the community meant that the authors were more likely to have explored diverse sociocultural context of these communities which could have accounted for the relatively high response rates [33]. 4)The diversity in terms of the location of the meetings (i.e. bars, social clubs, football matches, market places, and churches) may have reduced the selection bias and increased the response rate and coverage. Despite these strengths, it is very difficult to attribute the effect observed to any single type of community intervention mentioned within the complex intervention category. This is because the authors did not stratify the individual interventions and their effects. In addition, implementing these complex packages could be quite costly since they involve significant resource allocation in the form of personnel/providers. This means they may not be sustainable in resource poor settings which further highlights the need for authors to stratify the individual community strategies in the package to better inform heath programmes that may want to take up only certain community strategies based on resource constraints.

This systematic review and meta-analysis had several strengths and some limitations. The search was comprehensive because several databases and grey literature were searched and authors were contacted for any unpublished studies. There was also independent search and screening of articles by 2 reviewers which reduced bias. Unlike other systematic reviews on male partner involvement, the quality of studies included in our review was critically assessed. The heterogeneity was high when unadjusted odds ratios were used but adjusted odds ratios gave moderate heterogeneity. A limitation in the study was the high selection bias which resulted from the selection of a non-representative sample by the authors. This may limit the generalizability of the findings to certain groups of individuals. Despite this limitation, our study is the first meta-analysis to provide data on the impact of male involvement on the uptake of safe infant feeding practices and will therefore set the pace for more studies with improved recruitment of participants. Our study is also relevant in guiding policy and research in this very important area of male involvement in PMTCT. Another limitation is that no randomized trial has been conducted to investigate the impact of male involvement on the uptake of safe infant feeding practices. Even though randomised trials provide a higher level of evidence than cohort studies, the latter provide a more reliable picture of the real world than the former [34]. The findings from our study using cohort studies may therefore present a better picture of real world implementation science than if only randomized trials were used.

## Conclusion

Interventions aimed at promoting male partner involvement such as enhanced psychosocial interventions, verbal encouragement and complex community interventions increase the uptake of safe infant feeding options. The heterogeneity was moderate among studies.

### Recommendations for future research

No randomized controlled trial has been carried out to investigate the impact of male partner involvement on the uptake of safe infant feeding practices. Randomized controlled trials are needed to add to the strength evidence available. Stratification of the impact of each category of intervention is needed in future to further inform policy and research. Finally, an economic evaluation is needed to adequately inform policy.

## Supporting information

S1 Table PRISMA 2009 checklist. (PDF)

S2 Table Data extraction sheets. (PDF)

## Abbreviations

HIV: Human Immunodeficiency Virus
PMTCT: Prevention of Mother to Child Transmission
PRISMA: Preferred Reporting Items for Systematic Reviews and Meta-Analyses
RCT: Randomized Controlled Trial
SSA: Sub Saharan Africa
UNAIDS: Joint United Nations Programme on HIV/AIDS

## References

[1] UNAIDS. The Prevention Gap Report. 2016. http://www.unaids.org/sites/default/files/media_asset/2016-prevention-gap-report_en.pdf

[2] UNAIDS. Report on the Global Plan towards the elimination of new pediatric infection and keeping mothers alive. 2015. http://www.unaids.org/sites/default/files/media_asset/JC2774_2015ProgressReport_GlobalPlan_en.pdf

[3] UNAIDS. The Gap report. UNAIDS report on Global AIDS epidemic 2013. http://files.unaids.org/en/media/unaids/contentassets/documents/epidemiology/2013/gr2013/UNAIDS_Global_Report_2013_en.pdf.

[4] Ramjee G, Daniels B. Women and HIV in Sub-Saharan Africa. *AIDS Res Ther* 2013;10(1):30 10.1186/1742-6405-10-30 24330537 10.1186/1742-6405-10-30 PMC3874682

[5] World Health Organization: Guidelines on HIV and Infant Feeding 2010; Principles and Recommendations for Infant Feeding in the Context of HIV and a Summary of Evidence; 2010. http://whqlibdoc.who.int/publications/2010/9789241599535_eng.pdf. 24501786

[6] Lazarus R, Struthers H, Violari A. Promoting safe infant feeding practices Á the importance of structural, social and contextual factors in Southern Africa. *J Int AIDS Soc* 2013; 16:18037 10.7448/IAS.16.1.18037 23394899 10.7448/IAS.16.1.18037 PMC3568174

[7] Onono MA, Cohen CR, Jerop M, Bukusi EA, Turan JM. HIV serostatus and disclosure: implications for infant feeding practice in rural south Nyanza, Kenya. *BMC Public Health* 2014;14:390 10.1186/1471-2458-14-390 24754975 10.1186/1471-2458-14-390 PMC4041135

[8] Byamugisha R, Tumwine JK, Semiyaga N, Tylleskär T. Determinants of male involvement in the prevention of mother-to-child transmission of HIV programme in Eastern Uganda: A cross-sectional survey. *Reprod Health* 2010;7:12 10.1186/1742-4755-7-12 20573250 10.1186/1742-4755-7-12 PMC2913932

[9] Bedell RA, Van Lettow M, Landes M. Women’s choices regarding HIV testing, disclosure and partner involvement in infant feeding and care in a rural district of Malawi with high HIV prevalence. *AIDS Care* 2014;26:483–6. 10.1080/09540121.2013.841830 24090356 10.1080/09540121.2013.841830

[10] Ambia J, Mandala J. A systematic review of interventions to improve prevention of mother-to-child HIV transmission service delivery and promote retention. *J Int AIDS Soc* 2016;19(1):20309 10.7448/IAS.19.1.20309 27056361 10.7448/IAS.19.1.20309 PMC4824870

[11] Baiden F, Remes P, Baiden R, Williams J, Hodgson A, Boelaert M, et al Voluntary counseling and HIV testing for pregnant women in the Kassena-Nankana district of northern Ghana: is couple counseling the way forward? *AIDS Care* 2005;17:648–57. 10.1080/09540120412331319688 16036251 10.1080/09540120412331319688

[12] Aluisio A, Richardson BA, Bosire R, John-Stewart G, Mbori-Ngacha D, Farquhar C. Male Antenatal Attendance and HIV Testing Are Associated with Decreased Infant HIV Infection and Increased HIV Free Survival. *J Acquir Immune Defic Syndr* 2011;56:76–82. 10.1097/QAI.0b013e3181fdb4c4 21084999 10.1097/QAI.0b013e3181fdb4c4 PMC3005193

[13] Kiarie JN, Kreiss JK, Richardson BA, John-Stewart GC. Compliance with antiretroviral regimens to prevent perinatal HIV-1 transmission in Kenya. *AIDS* 2003;17:65–71. 10.1097/01.aids.0000042938.55529.e1 12478070 10.1097/01.aids.0000042938.55529.e1 PMC3387271

[14] Manjate Cuco RM, Munguambe K, Bique Osman N, Degomme O, Temmerman M, Sidat MM. Male partners’ involvement in prevention of mother-to-child HIV transmission in sub-Saharan Africa: A systematic review. *SAHARA J* 2015;12:87–105. 10.1080/17290376.2015.1123643 26726756 10.1080/17290376.2015.1123643

[15] Haile F, Brhan Y. Male partner involvements in PMTCT: a cross sectional study, Mekelle, Northern Ethiopia. *BMC Pregnancy Childbirth* 2014;14:65 10.1186/1471-2393-14-65 24521216 10.1186/1471-2393-14-65 PMC3923985

[16] Nyondo AL, Muula AS, Chimwaza AF. Assessment of strategies for male involvement in the prevention of mother-to-child transmission of HIV services in Blantyre, Malawi. *Glob Health Action* 2013;6:22780 10.3402/gha.v6i0.22780 24345635 10.3402/gha.v6i0.22780 PMC3866839

[17] Moher D, Liberati A, Tetzlaff J, Altman DG, Altman D, Antes G, et al Preferred reporting items for systematic reviews and meta-analyses: The PRISMA statement. *PLoS Med* 2009;6. PMC3090117 21603045

[18] Takah NF, Kennedy ITR, Johnman C. Impact of approaches in improving male partner involvement in the prevention of mother-to-child transmission (PMTCT) of HIV on the uptake of PMTCT services in sub-Saharan Africa: a protocol of a systematic review and meta-analysis. *BMJ open* 2016;6:e012224 10.1136/bmjopen-2016-012224 27371555 10.1136/bmjopen-2016-012224 PMC4947788

[19] DeLuca BJ, Mullins MM, Lyles CM, Crepaz N, Kay L, Thardiparthi S.Developing a Comprehensive Search Strategy for Evidence Based Systematic Reviews. *Evid Based Libr Inf Pract* 2010;3:(1).

[20] Higgins JPT, Thompson SG, Deeks JJ, Altman DG. Measuring inconsistency in meta-analyses. *BMJ* 2003;327(7414):557–60. 10.1136/bmj.327.7414.557 12958120 10.1136/bmj.327.7414.557 PMC192859

[21] Wells GA, Shea B, O’Connell D, Peterson J, Welch V, Losos M et al. The Newcastle-Ottawa scale (NOS) for assessing the quailty of nonrandomised studies in meta-analyses. http://www.ohri.ca/programs/clinical_epidemiology/oxford.htm 2009 Feb 1. 2009;2009.

[22] Kalembo FW, Zgambo M, Mulaga AN, Yukai D, Ahmed NI. Association between male partner involvement and the uptake of prevention of mother-to-child transmission of HIV (PMTCT) interventions in Mwanza district, Malawi: a retrospective cohort study. *PLoS One* 2013;8(6):e66517 10.1371/journal.pone.0066517 23776683 10.1371/journal.pone.0066517 PMC3680434

[23] Msuya SE, Mbizvo EM, Hussain A, Uriyo J, Sam NE, Stray-Pedersen B. Low male partner participation in antenatal HIV counselling and testing in northern Tanzania: implications for preventive programs. *AIDS Care* 2008;20:700–9. 10.1080/09540120701687059 18576172 10.1080/09540120701687059

[24] Brou H, Djohan G, Becquet R, Allou G, Ekouevi DK, Viho I, et al When Do HIV-Infected Women Disclose Their HIV Status to Their Male Partner and Why? A Study in a PMTCT Programme, Abidjan. *PLoS Med* 2007;4(12):e342 10.1371/journal.pmed.0040342 18052603 10.1371/journal.pmed.0040342 PMC2100145

[25] Farquhar C, Kiarie JN, Richardson BA, Kabura MN, John FN, Nduati RW, et al Antenatal couple counseling increases uptake of interventions to prevent HIV-1 transmission. *J Acquir Immune Defic Syndr* 2004; 37(5):1620–6. 15577420 10.1097/00126334-200412150-00016 PMC3384734

[26] Semrau K, Kuhn L, Vwalika C, Kasonde P, Sinkala M, Kankasa C, et al Women in couples antenatal HIV counseling and testing are not more likely to report adverse social events. *AIDS* 2005;19:603–9. 15802979 10.1097/01.aids.0000163937.07026.a0 PMC1201374

[27] Brusamento S, Ghanotakis E, Tudor Car L, van-Velthoven MH, Majeed A, Car J. Male involvement for increasing the effectiveness of prevention of mother-to-child HIV transmission (PMTCT) programmes. *Cochrane Database Syst Rev* 2012;10:CD009468 10.1002/14651858.CD009468.pub2 23076959 10.1002/14651858.CD009468.pub2 PMC6718228

[28] Yargawa J, Leonardi-bee J. Male involvement and maternal health outcomes: systematic review and meta-analysis. *J Epidemiol Community Health* 2015; 69(6):604–12. 10.1136/jech-2014-204784 25700533 10.1136/jech-2014-204784 PMC4453485

[29] Auvinen J, Kylma J, Suominen T. Male involvement and prevention of mother-to-child transmission of HIV in Sub-Saharan Africa: an integrative review. *Curr HIV Res* 2013;11(2):169–77. 23432492 10.2174/1570162x11311020009

[30] Morar NS, Naidoo S, Goolam A, Ramjee G. Research participants’ skills development as HIV prevention peer educators in their communities. *J Health Psychol* 2018; 23(10):1343–1349. 10.1177/1359105316655470 27371447 10.1177/1359105316655470

[31] Medley A, Garcia-moreno C, Mcgill S, Maman S. Rates, barriers and outcomes of HIV serostatus disclosure among women in developing countries: implications for prevention of mother-to-child transmission programmes. *Bull World Health Organ* 2004;82(4):299–307. 15259260 PMC2585956

[32] Mbonu NC, Van DenBorne B, De Vries NK. Stigma of People with HIV / AIDS in Sub-Saharan Africa: A Literature Review. *J Trop Med* 2009;2009:145891 10.1155/2009/145891 20309417 10.1155/2009/145891 PMC2836916

[33] Busza J, Walker D, Hairston A, Gable A, Pitter C, Lee S, et al Community-based approaches for prevention of mother to child transmission in resource-poor settings: a social ecological review. *J Int AIDS Soc* 2012; 15 Suppl 2:17373. 22789640 10.7448/IAS.15.4.17373 PMC3499910

[34] Besen J, Gan SD. A critical evaluation of clinical research study designs. *J Invest Dermatol* 2014;134:e18. 24518116 10.1038/jid.2013.545

