# Supplementary material for: The impact of approaches in improving male partner involvement in the prevention of mother-to-child transmission of HIV on the uptake of safe infant feeding practices by HIV positive women in sub-Saharan Africa. A systematic review and meta-analysis
Source: PLoS One. 2018 Dec 3;13(12):e0207060. doi: 10.1371/journal.pone.0207060 (PMC6277072; doi:10.1371/journal.pone.0207060)
Supplement: S2 Table — (PDF) [file pone.0207060.s002.pdf]

S2A Table. Data extraction sheet 1.General data items for all studies

Author	Country	Year of study	Study design	Study setting	Sample size	Approach used	LCIOR	OR	UCIOR
**Aluiso et al**	Kenya	2011	Cohort	Urban	456	Verbal encouragement	1.02	1.59	2.44
**Brou et al**	Ivory Coast	2007	Cohort	Urban	546	Complex community interventions	1.04	1.54	2.27
**Farquhar et al**	Kenya	2004	Cohort	Urban	122	Enhanced psychosocial intervention	1.08	5.1	24.05
**Kalembo et al**	Malawi	2013	Cohort	Rural	476	Verbal encouragement	1.6	3.1	6.2
**Msuya et al**	Tanzania	2007	Cohort	Urban	184	Enhanced psychosocial intervention	2.18	5.15	12.16
**Semrau et al**	Zambia	2005	Cohort	Urban	2141	Complex community interventions	3.74	4.8	6.17

LCIOR=Lower confidence interval of odds ratio; OR=unadjusted Odds Ratio; UCIOR=Upper confidence interval of odds ratio

S2B Table. Data extraction sheet 2.Data items with adjusted ORs only

Author	Country	Year of study	Study design	Study setting	Sample size	Approach used	LCIOR	AdjOR	UCIOR
**Aluiso et al**	Kenya	2011	Cohort	Urban	456	Verbal encouragement	1.02	1.59	2.44
**Brou et al**	Ivory Coast	2007	Cohort	Urban	546	Complex community interventions	1.04	1.54	2.27
**Farquhar et al**	Kenya	2004	Cohort	Urban	122	Enhanced psychosocial intervention	1.08	5.1	24.05
**Msuya et al**	Tanzania	2007	Cohort	Urban	184	Enhanced psychosocial intervention	2.05	5.8	16.36

LCIOR=Lower confidence interval of odds ratio; AdjOR=Adjusted Odds Ratio; UCIOR=Upper confidence interval of odds ratio

S2C Table. Data extraction sheet 3. Number of participants from each study to permit calculation of ORs

Author	Number of women taking up safe infant feeding practice with male involvement	Number of women not taking up safe infant feeding practices with male involvement	Total number of women with male involvement	Number of women taking up safe infant feeding practices without male involvement	Number of women not taking up safe infant feeding practices without male involvement	Number of women without male involvement	Sample size
**Farquhar et al**	3	5	8	12	102	114	122
**Semrau et al**	211	100	311	559	1271	1830	2141
